# An investigation into the mechanism for Kaempferol improving melanocyte death based on network Pharmacology and experimental verification

**DOI:** 10.1038/s41598-025-91905-0

**Published:** 2025-03-12

**Authors:** Jinming Li, Yeqiang Song, Meng Yang

**Affiliations:** 1 Department of Dermatology, Shandong Provincial Third Hospital, Jinan, 250000 China; 2 Cosmetic dermatology, Affiliated Hospital of Shandong University of Traditional Chinese Medicine, Jinan, 250000 China

**Keywords:** Kaempferol, Ferroptosis, Vitiligo, GPX4, Skin diseases, Drug delivery

## Abstract

Melanocyte (MC) death represents the basic pathological change of vitiligo. Kaempferol (Kae) is one of the main active ingredients of Tribulus terrestris, which is a commonly used Chinese medicine in the treatment of vitiligo. However, it remains unclear whether Kae can improve MC death, and hence relevant mechanisms need to be further explored. Therefore, we aimed to investigate the effect of Kae on MC death and relevant mechanisms. The targets of Kae and the differential genes of vitiligo were screened based on different databases. Besides, the protein-protein interaction (PPI) network of the common target of Kae and vitiligo was constructed to further identify the “keycluster” genes of the drug-disease interaction (DDI) network. In addition, the enrichment analysis based on Gene Ontology (GO), Disease Ontology (DO), and Kyoto Encyclopedia of Genes and Genomes (KEGG) was performed on the “keycluster” genes. Based on the network pharmacological results, it was found that Kae may ameliorate MC death through the ferroptosis pathway. Hence, the ferroptosis model of human primary epidermal melanocyte 1 (HEM-1) was induced by RAS-selective lethal 3 (RSL3) and then co-cultured with Kae. Moreover, the role of Kae in MC ferroptosis was investigated by detecting the changes in mitochondrial morphology and functions, the levels of reactive oxygen species (ROS) and iron ions, the protein expression of glutathione peroxidase 4 (GPX4), and antioxidant activities. Finally, si-GPX4 was used to silence the ferroptosis core protein GPX4 to re-examine the above indicators, thus verifying relevant mechanisms. The network pharmacology results showed that Kae was responsive to oxidative stress and ROS. The treatment of vitiligo by Kae mainly involved pigmentation, melanin metabolic processes, and such signaling pathways as melanogenesis, ferroptosis, and tyrosine metabolism. The in vitro experiment results indicated that Kae can effectively improve RSL3-induced HEM-1 ferroptosis, including alleviating mitochondrial damage, decreasing the level of ROS and iron ions, and up-regulating the expression of GPX4 and antioxidants. After silencing GPX4, the protective effect of Kae against HEM-1 ferroptosis was attenuated. Our study concluded that Kae can reduce RSL3-induced ferroptosis in HEM-1, and its mechanism is related to the regulation of the expression of the ferroptosis pathway protein GPX4. These findings are expected to provide novel insights into the treatment of vitiligo.

## Introduction

Vitiligo is an autoimmune disease of the skin that targets pigment-producing Melanocytes (MCs) and results in patches of depigmentation that are visible as white spots^1,2^. Although the pathogenesis of vitiligo is not well understood, various theories have been suggested for the cause of melanocyte loss in vitiligo; Genetics, environment, and autoimmunity are considered to be the main factors, with strong evidence supporting them^1^. Because of an inherited inability to manage stressors from normal cellular processes (e.g., melanogenesis) or exposure to environmental factors (injury or chemicals), reactive oxygen species (ROS) are released from melanocytes. ROS play a crucial role in the pathogenesis of vitiligo and are the primary cause of immune inflammation. On one hand, ROS promote the release of inflammatory cytokines, such as interleukin (IL)-1β and IL-18, and activate innate immune cells. On the other hand, ROS can initiate a signaling cascade through the unfolded protein response, which leads to the direct release of pro-inflammatory cytokines, including IL-6 and IL-8, and antagonizes the suppressor function of regulatory T cells^3^. These effects ultimately result in melanocyte dysfunction or death, thereby contributing to the development of vitiligo.

Melanocytes can undergo cell death through various mechanisms. One such mechanism involves ROS, which can lead to an increase in intracellular iron ions. This accumulation of iron ions subsequently promotes the formation of lipid peroxides and activates the ferroptosis pathway^4,5^. Ferroptosis, an iron-dependent mode of programmed cell death, is typically associated with substantial iron accumulation, which can lead to the production of excess ROS through the Fenton reaction, thereby exacerbating oxidative damage^6,7^. Morphologically, cells undergoing ferroptosis exhibit characteristics such as plasma membrane blistering, reduction or disappearance of mitochondrial ridges, normal nuclear size, and insufficient chromatin condensation^8^. Glutathione peroxidase 4 (GPX4) has been identified as a core regulator and biomarker of ferroptosis^9^. It has been corroborated in many studies that GPX4 decreases in both the serum and tissue of patients with vitiligo^10^. RAS-selective lethal 3 (RSL3) is a classical inducer of cell ferroptosis^11,12^. In our previous studies, we have established a ferroptosis model of HEM-1 using RSL3^5^. RSL3 directly inhibits the catalytic selenocysteine in GPX4, which prevents the elimination of polyunsaturated fatty acid (PUFA) peroxide, thus inhibiting the synthesis of glutathione (GSH). GSH depletion leads to the inactivation of GPX4 and the activation of lipoxygenase, ultimately resulting in ferroptosis^13^.

According to traditional Chinese medicine, tribulus terrestris is often used in the treatment of vitiligo. Kaempferol (Kae) is a flavonoid compound and one of the main components of Tribulus Tribulus, it has a wide range of anti-inflammatory and antioxidant effects^14^. Previous studies have shown that kaempferol can reduce the production of ROS in melanocytes, but its effect on melanocyte death was unclear^15^. Based on network pharmacological studies, we found that Kae may take effect in the treatment of vitiligo by the melanocyte ferroptosis process. Previous studies have also shown, Kae can regulate the mechanism of ferroptosis ferroptosis in neurons and liver cells^16–18^. In this study, the ferroptosis model of MCs was constructed by RSL3, and the effect of Kae on the ferroptosis of MCs was explored, so as to explore the effect and mechanism of kaempferol in improving melanocyte death.

## Results

### Screening and analysis of targets related to Kae effects

**Fig. 1:**
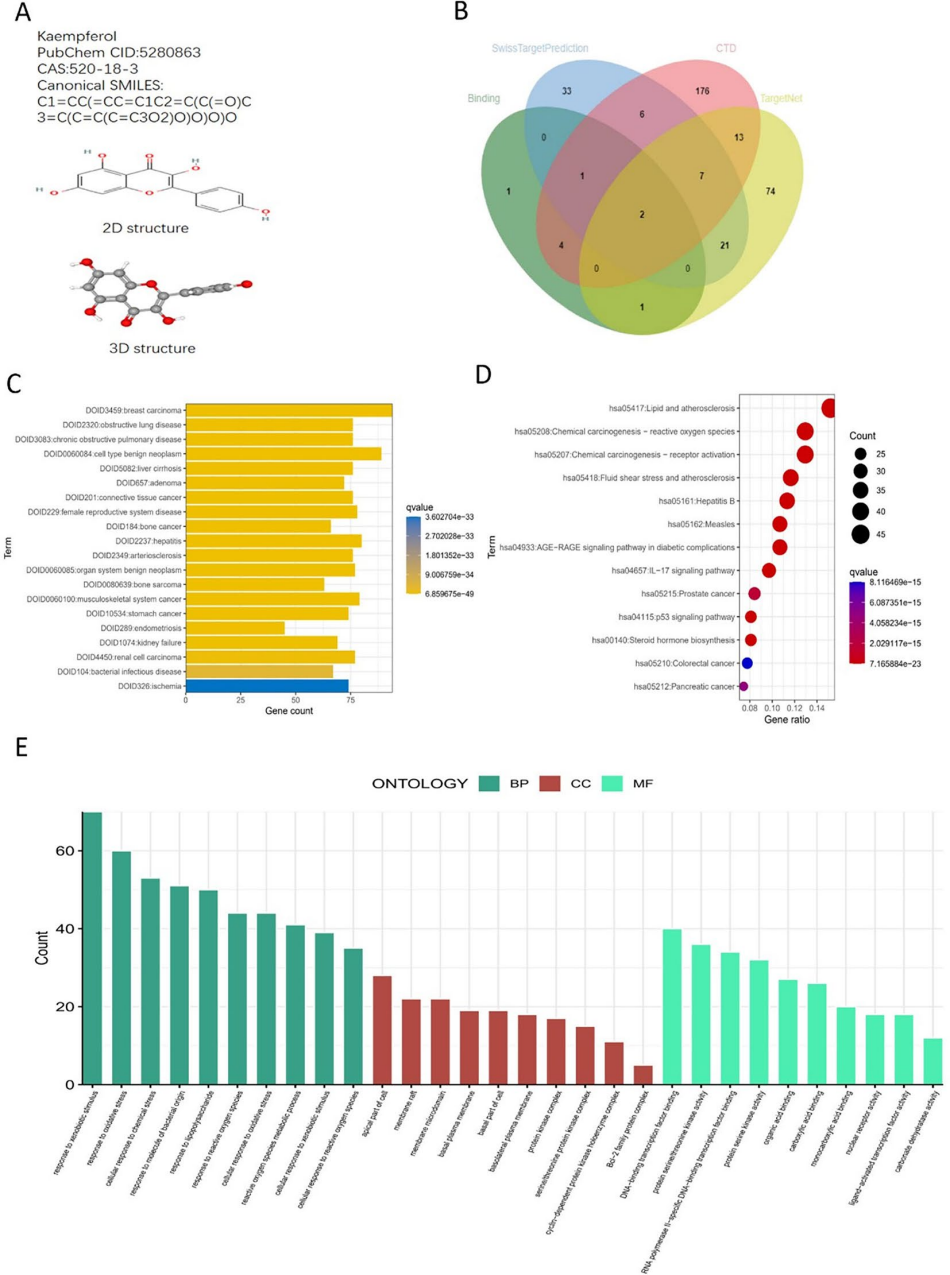
Drug information and target enrichment analysis. (**A**) Overview of kae and its two-dimensional (2D) and three-dimensional (3D) structures. (**B**) The targets of kae were screened using SwissTargetPrediction, CTD, BindingDB, and TargetNet, resulting in the creation of a Venn diagram. (**C**) DO enrichment analysis of potential drug targets; the vertical axis represents specific classifications, while the horizontal axis indicates the number of genes enriched within each specific entry. (**D**) Bubble map illustrating KEGG enrichment analysis for potential drug targets. The transition from blue to red indicates an increasing significance of the q-value, with the diameter of each bubble representing the number of genes associated with each pathway. (**E**) GO enrichment analysis of potential drug targets; the horizontal axis represents specific classifications, and the vertical axis indicates the number of genes enriched within each specific entry.

The two-dimensional (2D) and three-dimensional (3D) structures and related information of Kae were obtained from PubChem (Pub Chem CID: 5280863; CAS: 5280863). After the targets from SwissTargetPrediction, CTD, BindingDB, and TargetNet were screened, a total of 339 targets were obtained (Fig. 1A and B).

## Enrichment analysis of targets related to Kae effects

The top 10 items were selected to plot the DO, KEGG, and GO enrichment analysis charts. The focus of the DO enrichment analysis was placed on gene enrichment related to the disease, where the count value of vitiligo was 18 (Fig. 1C). The KEGG enrichment analysis results showed that Kae can fulfill functions mainly through ROS and other signaling pathways (Fig. 1D). GO is subdivided into three parts: cellular component (CC), molecular function (MF), and biological process (BP). The GO enrichment analysis results showed that Kae was related to such BPs as response to oxidative stress and response to ROS, such CCs as the basal part of cells and membrane microdomain, and such MFs as protein serine/threonine kinase activity, iron ions binding, and intracellular iron ions homeostasis (Fig. 1E).

### Differential gene analysis of vitiligo

**Fig. 2:**
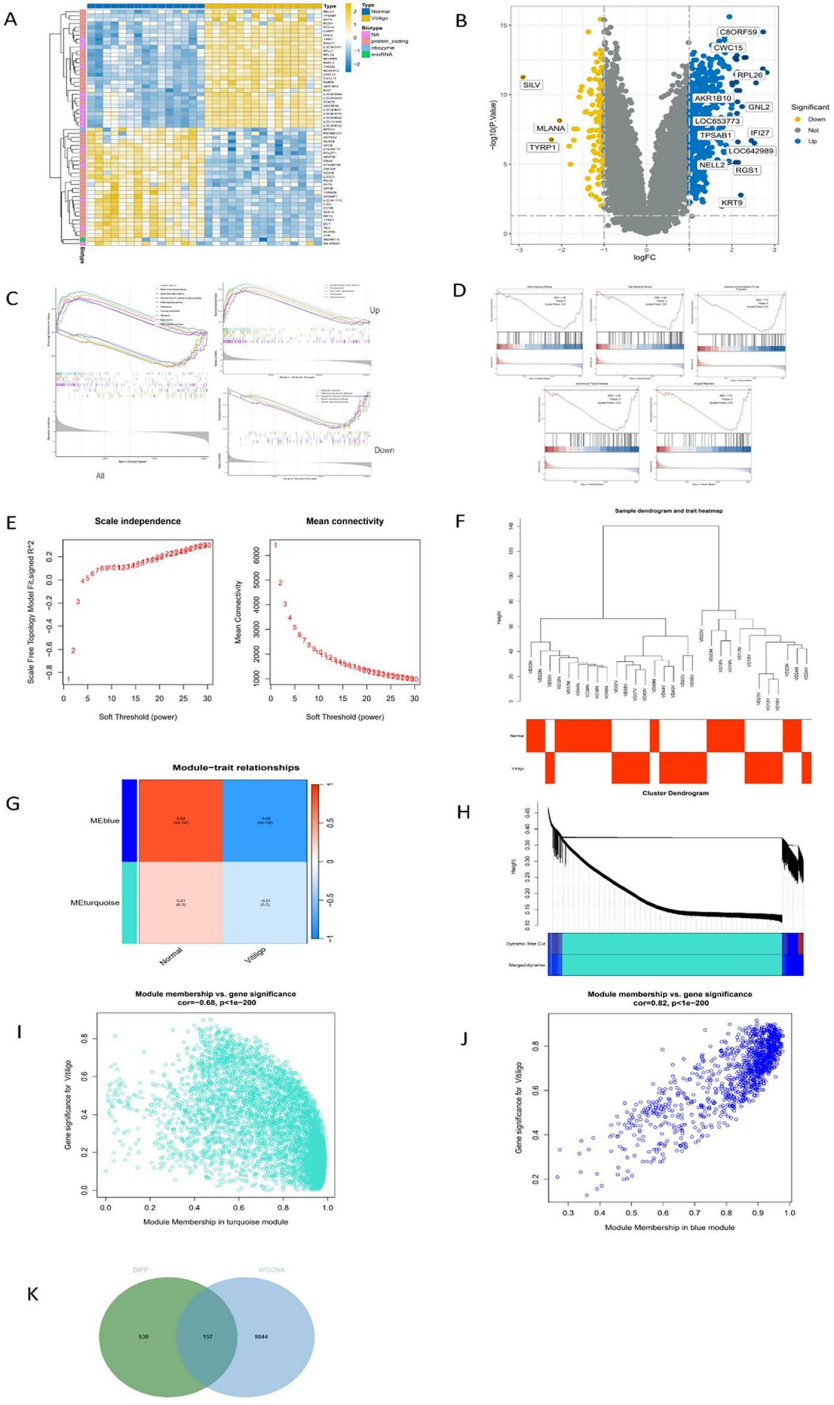
Analysis of differentially expressed genes between vitiligo subjects and healthy subjects. (**A**) The heat map illustrates the differentially expressed genes between subjects with vitiligo and healthy subjects. The horizontal axis represents the samples, categorized into two groups: normal and vitiligo. The vertical axis represents the genes, where yellow indicates high expression and blue indicates low expression. (**B**) The volcano plot depicts the differentially expressed genes between vitiligo subjects and healthy subjects. The horizontal axis represents the fold change in gene expression after comparing the normal group to the vitiligo group, with a logarithmic transformation applied. The vertical axis represents the corrected p-value, expressed as -log10. Each dot corresponds to a detected gene, with blue and yellow denoting up-regulated and down-regulated genes, respectively, while gray indicates genes that are not differentially expressed. (**C** and **D**) C: All GSEA enrichment analysis. D: Down GSEA enrichment analysis. The GSEA diagram is divided into three parts: top, middle and bottom. The first section is the Enrichment score (ES) line chart, showing how the ES value is represented at each location when the analysis is calculated sequentially along the sequenced gene set. The third section depicts the distribution of rank values for all genes following sequencing. The genes represented by the red areas of the heat map are highly expressed in the normal group, whereas the genes indicated by the blue areas are highly expressed in the vitiligo group. The Signal-to-Noise ratio for each gene, calculated using the previously selected sequencing method, is illustrated in the gray area of the diagram. (**E**) R^2^ values corresponding to various soft thresholds and gene adjacency coefficients across different soft thresholds are presented. The horizontal axes of the left and right graphs indicate the values of power. In the left graph, the vertical axis represents the scale-free fit index (signed R^2^); a higher squared correlation coefficient suggests that the network more closely resembles a scale-free distribution. Conversely, the vertical axis of the right graph depicts the average number of connections for all nodes, with lower values being preferable. (**F**) A hierarchical clustering tree is shown. (**G**) A gene tree map constructed through hierarchical clustering of gene adjacency coefficients is displayed. The horizontal axis represents traits (one for Normal and the other for vitiligo), while the vertical axis corresponds to the respective module, denoted by the eigengene of each module. This diagram includes two modules, marked in blue and turquoise. The grid illustrates the correlation between phenotypic traits on the horizontal axis and each module on the vertical axis, alongside the p-value. A deeper yellow indicates a stronger positive correlation, while a darker blue signifies a stronger negative correlation. The legend on the far right indicates the correlation scale, where values closer to 1 are represented in darker yellow (indicating positive correlation) and values closer to -1 are depicted in darker blue (indicating negative correlation). (**H**) The relationship between normal gene modules and vitiligo characteristics is illustrated in the figure. The top half displays a hierarchical clustering tree of genes, while the bottom half depicts the gene modules. Genes that are closer together are grouped into the same module. The color ‘Merged’ indicates modules that have been combined, with each color representing a distinct module; gray signifies that a gene does not belong to any module. (**I**,**J**) The turquoise module exhibited a significant negative correlation with vitiligo, whereas the blue module showed a significant positive correlation. The scatter plot of the module membership (MM) and gene significance (GS) correlation illustrates the relationship between these two metrics. The data indicate a strong correlation between GS and MM (cor=-0.68, *p* < 1e − 200; cor = 0.82, *p* < 1e − 200). (K) A Venn diagram represents the intersection of differentially expressed genes (DIFF) and those identified through WGCNA.

A total of 30 samples were included in the study, including 15 affected skin samples (the vitiligo group) and 15 normal skin samples (the normal group). By comparing the vitiligo-related genes between the control group and the experimental group, a total of 7,357 differential genes were identified, including 3,390 up-regulated genes and 3,967 down-regulated genes. In the heat map, different colors represent different gene expression levels. The yellower the color, the higher the gene expression; the bluer the color, the lower the gene expression. White represents the genes with intermediate expression (Fig. 2A). In the volcano plot, each dot represents a gene. Specifically, the yellow dot on the left represents the down-regulated gene with log FC<-1, the gray dot in the middle represents the gene with no difference between the vitiligo group and the normal group, and the blue dot on the right represents the up-regulated gene with log FC > 1 (Fig. 2B). The results showed that there were significant differences in gene expression between the vitiligo group and the normal group.

### GSEA

The GSEAwas performed based on GSE75819 data to explore the differential biological function between healthy subjects and vitiligo patients. The results showed that the high-expression group was mainly involved in such BPs as basal transcription factors, pyruvate metabolism, and proteasome and oxidative phosphorylation (Fig. 2C). Meanwhile, 5 GSEA maps of the low-expression group were obtained. It was found that the low-expression group was mainly involved in the VEGF signaling pathway, Notch signaling pathway, intestinal immune network for IgA production, and other pathways (Fig. 2D).

### Co-expression network construction based on the WGCNA

The “pickSoftThreshold” function was used to raise the soft threshold value to calculate the scale-free topological fitting index and average connectivity. Consequently, the soft threshold value of 14 and the minimum module value of 30 were used to define the adjacency matrix (Fig. 2E). It can be seen from the sample clustering tree that all samples participated in the construction of the network (Fig. 2F). In the gene tree map constructed by hierarchical clustering of the gene adjacency coefficient, the correlation between normal genes and vitiligo genes was grouped into two modules, namely the blue module and the turquoise module. The above two modules were significantly correlated with vitiligo phenotypes (*P* ≤ 0.05). In the blue module, 1,186 genes were positively correlated with vitiligo genes; in the turquoise module, 8,814 genes were negatively correlated with vitiligo genes (Fig. 2G and H). To further confirm the correlation between each module and vitiligo, the correlation coefficient of each module was calculated. The results showed that the turquoise module was significantly negatively correlated with vitiligo (cor=-0.68, *P* < 0.05) (Fig. 2I), while the blue module was significantly positively correlated with vitiligo (cor = 0.82, *P* < 0.05) (Fig. 2J). The genes obtained from the DIFF and WGCNA were intersected to plot the Wayne diagram, and a total of 10,530 genes were obtained (Fig. 2K).

### Drug-disease “MCODE” key cluster network and functional enrichment

**Fig. 3:**
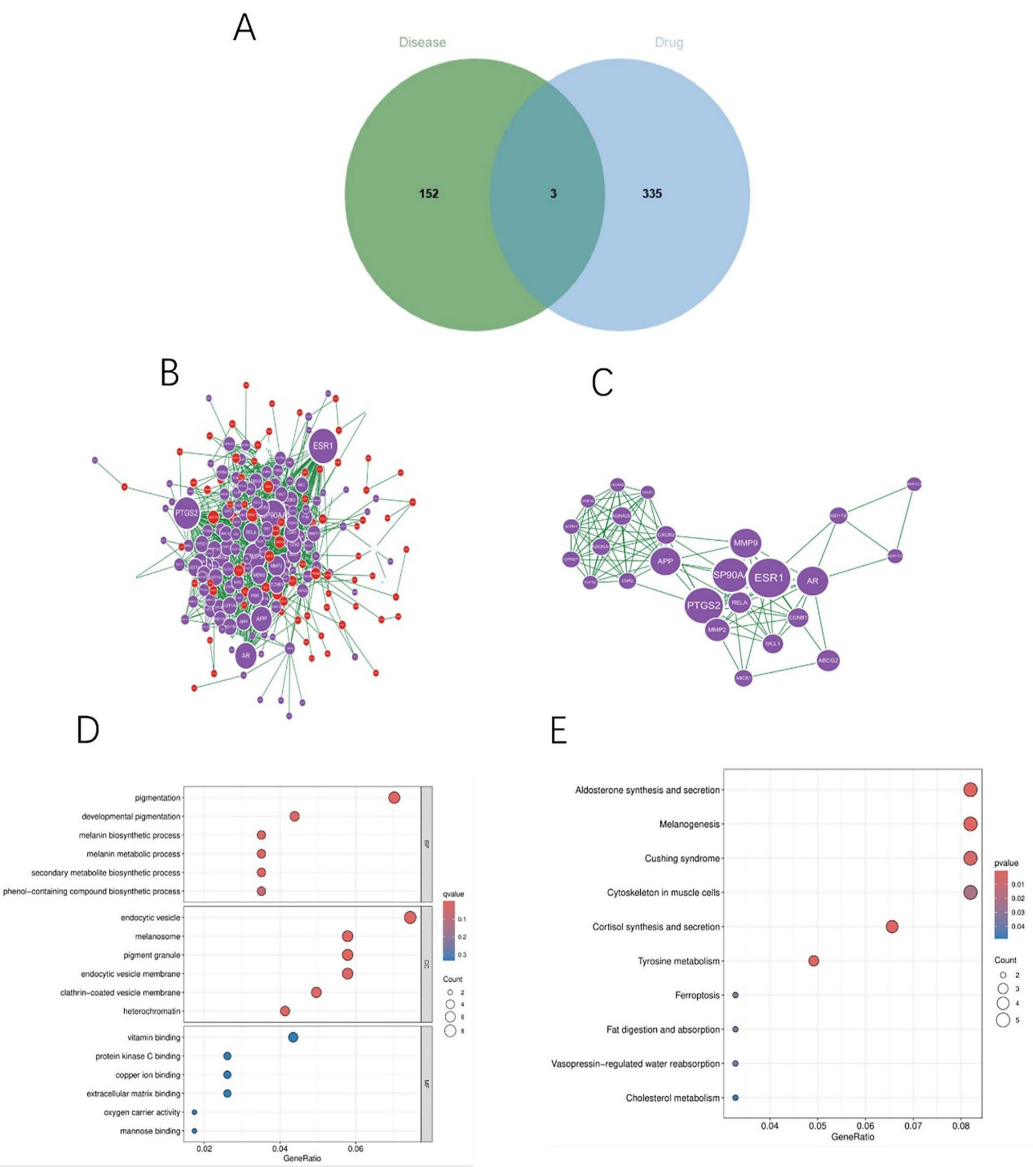
Analysis of PPI genes and keycluster genes. (**A**) Venn diagram illustrating the intersection of disease genes and drug target genes. (**C**) PPI network of the intersecting genes. Each node represents a protein, with the letter marked on the node corresponding to the gene symbol; the lines between the nodes indicate interactions between the proteins. (**D**) PPI network of key cluster genes. (**E**) GO enrichment analysis of key cluster genes. The horizontal axis represents specific classifications, while the vertical axis indicates the number of genes enriched within each classification. (**F**) KEGG enrichment analysis of key cluster genes. The transition from blue to red signifies an increasing significance of the q-value, with the diameter of each bubble representing the number of genes associated with each pathway.

After the duplicate genes in vitiligo disease genes and drug target genes were removed, the Wynn diagram (Fig. 3A) was plotted. The PPI network diagram was constructed, and the central size of the DDI network was obtained using the CytoNCA plug-in in Cytoscape (Fig. 3B). The gene network subgroup with the highest score was selected through the MCODE plug-in, and there were 25 keycluster genes in this subgroup (Fig. 3C). The GO enrichment analysis results showed that Kae in the treatment of vitiligo was mainly related to such BPs as pigmentation and melanin metabolic process, such CCs as melanosome and pigment granule, and such MFs as oxygen carrier activity (Fig. 3D). The KEGG enrichment analysis results showed that the treatment of vitiligo with Kae was mainly related to such signaling pathways as melanogenesis, ferroptosis, and tyrosine metabolism (Fig. 3E).

### Kae prevented RSL3-induced growth Inhibition in MCs

**Fig. 4:**
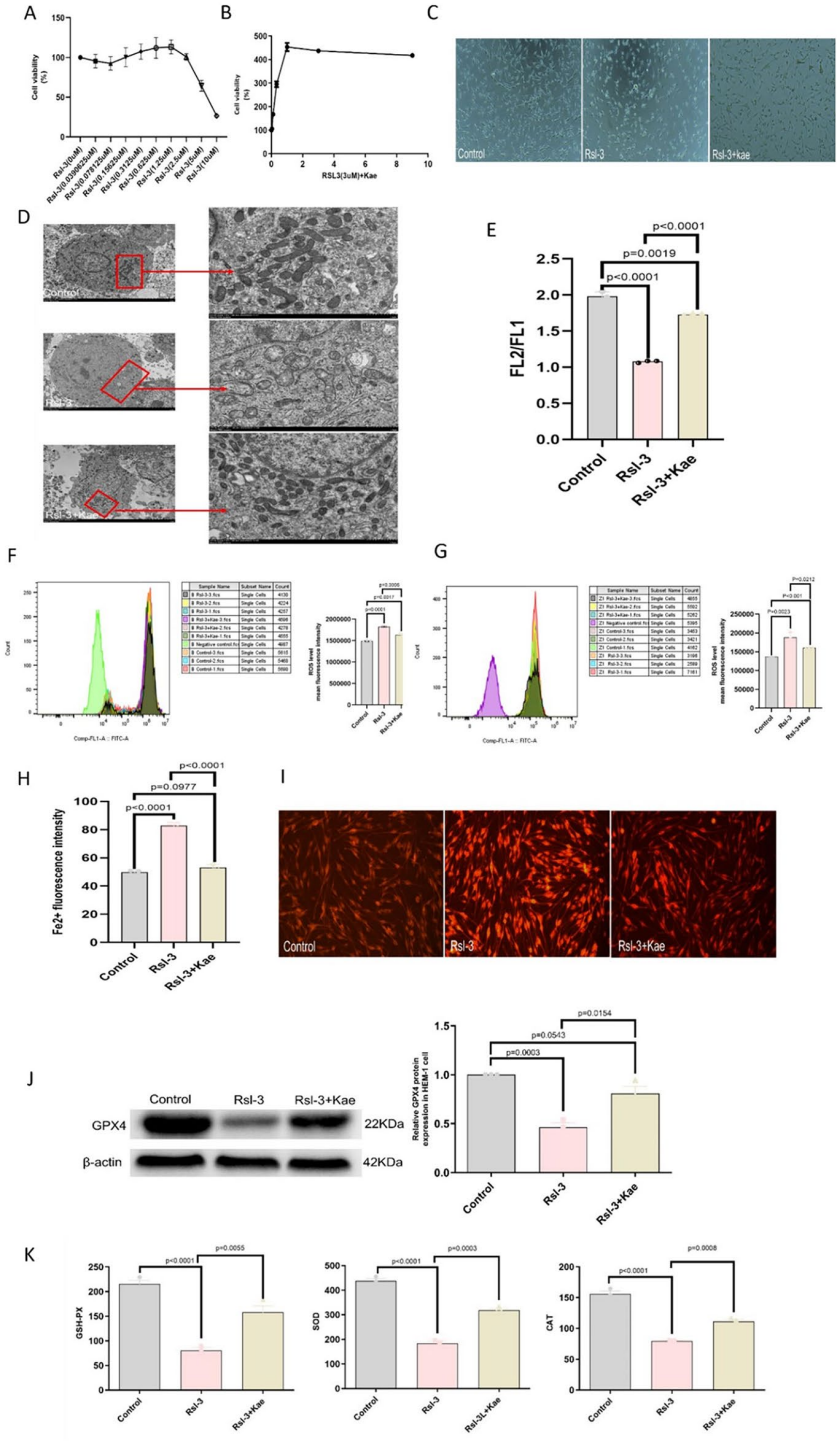
Kae interfered with the ferroptosis of HEM-1. (**A**) Damage to HEM-1 by different concentrations of RSL3. (**B**) Protective effects of Kae on the ferroptosis of HEM-1. (**C**) The number of HEM-1 in each group under a microscope. (**D**) Mitochondrial morphological changes of HEM-1 treated with RSL3, Kae, and their combination for 24 h. (**E**) Changes in the membrane potential in HEM-1 treated by RSL3, Kae, and their combination for 24 h. (**F**) The production of intracellular ROS was detected by FC. (**G**) The production of lipid ROS was detected by FC. (**H**) Changes in ferroptosis in HEM-1 treated with RSL3 and Kae. (I) The accumulation of ferroptosis in cells was detected by fluorescence microscopy. (**J**) WB analysis of GPX4, a key protein of ferroptosis. (**K**) Activities of antioxidant enzymes (GSH Px, SOD, and CAT).

After the cells were treated with RSL3 at a certain concentration gradient for 24 h, the cell viability was tested by the CCK-8 assay. Finally, the concentration of RSL3 was determined to be 3µM for subsequent procedures (Fig. 4A). To analyze the effects of Kae on HEM-1 treated with RSL3, the experiment was divided into three groups: the control group, the RSL3 group, and the Kae + RSL3 group. Specifically, Kae was cultured at a series of concentration gradients for 2 h, starting with a concentration of 9 µM, followed by a 1/3 dilution across ten wells. Subsequently, RSL3 was added at a concentration of 3 µM for incubation for 24 h. The cell viability was measured by the CCK-8 assay. The results indicated that Kae at 1 µM significantly reversed the inhibition of MC growth induced by RSL3 at a concentration of 3 µM(Fig. 4B). Under an electron microscope, the number of cells treated with RSL3 decreased significantly compared with the control group. The number of HEM-1 in the RSL3 + Kae group was higher than that in the RSL3 group (Fig. 4C).

### Kae improved mitochondrial morphology and dysfunction

Through transmission electron microscopy, it was observed that the mitochondrial volume of HEM-1 in the RSL3 group decreased, the density of the double-layer membrane increased, the mitochondrial outer membrane was broken, and the mitochondrial ridge disappeared. After Kae intervention, the morphological damage of mitochondria was significantly alleviated (Fig. 4D). Meanwhile, mitochondrial membrane potential depolarization was relieved after Kae treatment (*P* < 0.0001) (Fig. 4E). These results indicated that Kae could ameliorate mitochondrial morphological changes and mitochondrial dysfunction induced by RSL3 in HEM-1.

### Kae reduced ROS and iron ions levels in RSL3-treated HEM-1

Under the induction of RSL3, HEM-1 cells produced a large number of ROS, resulting in oxidative stress damage. To prove whether Kae can exert antioxidant effects, the content of ROS produced in HEM-1 cells was measured before and after treatment. The experimental results showed that the levels of both intracellular and lipid ROS in the RSL3 group were significantly higher than those in the control group (*P* < 0.01), while the levels of intracellular and lipid ROS in the Kae group were significantly lower than those in the RSL3 group (*P* < 0.01) (Fig. 4F-G). These results suggested that Kae exerted a protective effect on reducing intracellular and lipid ROS levels.

Meanwhile, the experimental results showed that compared with the control group, there was a large amount of iron ions accumulation in the cells of the RSL3 group (*P* < 0.0001), and the accumulation of iron ions could be significantly inhibited after Kae treatment (*P* < 0.0001) (Fig. 4H). Meanwhile, the accumulation of iron ions in the cells was detected under a fluorescence microscope. The results showed that compared with the control group, RSL3 treatment resulted in brighter and more fluorescence accompanied by the accumulation of a large amount of Fe^2+^. Besides, the accumulation amount of Fe^2+^ in the RSL3 + Kae group was lower than that in the RSL3 group (Fig. 4I). Therefore, these results suggested that Kae may reduce intracellular and lipid ROS levels by interacting with iron ions.

### Kae protected HEM-1 from ferroptosis by up-regulating GPX4 protein expression

To verify the effect of Kae on the ferroptosis of HEM-1, WB was used to detect the expression of GPX4. The WB results showed that GPX4 protein expression in the RSL3 group was down-regulated compared with the control group (*P* = 0.0003). The GPx4 protein expression level in the Kae group was higher than that in the RSL3 group (*P* = 0.0154) (Fig. 4J). These results suggested that RSL3 down-regulated the expression of GPX4, and Kae may up-regulate the expression of GPX4, thus playing an anti-ferroptosis role in MCs.

### Kae increased antioxidant activities

In this experiment, the activities of superoxide dismutase (SOD), (catalase) CAT, and (glutathione peroxidase) GSH Px were detected by corresponding kits. It was found that compared with the control group, the activities of SOD, CAT, and GSH Px in the RSL3 group decreased (*P* < 0.0001). Besides, the activities of three antioxidant enzymes in the Kae group were higher than those in the RSL3 group (*P* < 0.001) (Fig. 4K).

### Kae alleviated mitochondrial damage and cell proliferation through the GPX4 ferroptosis signaling pathway

**Fig. 5:**
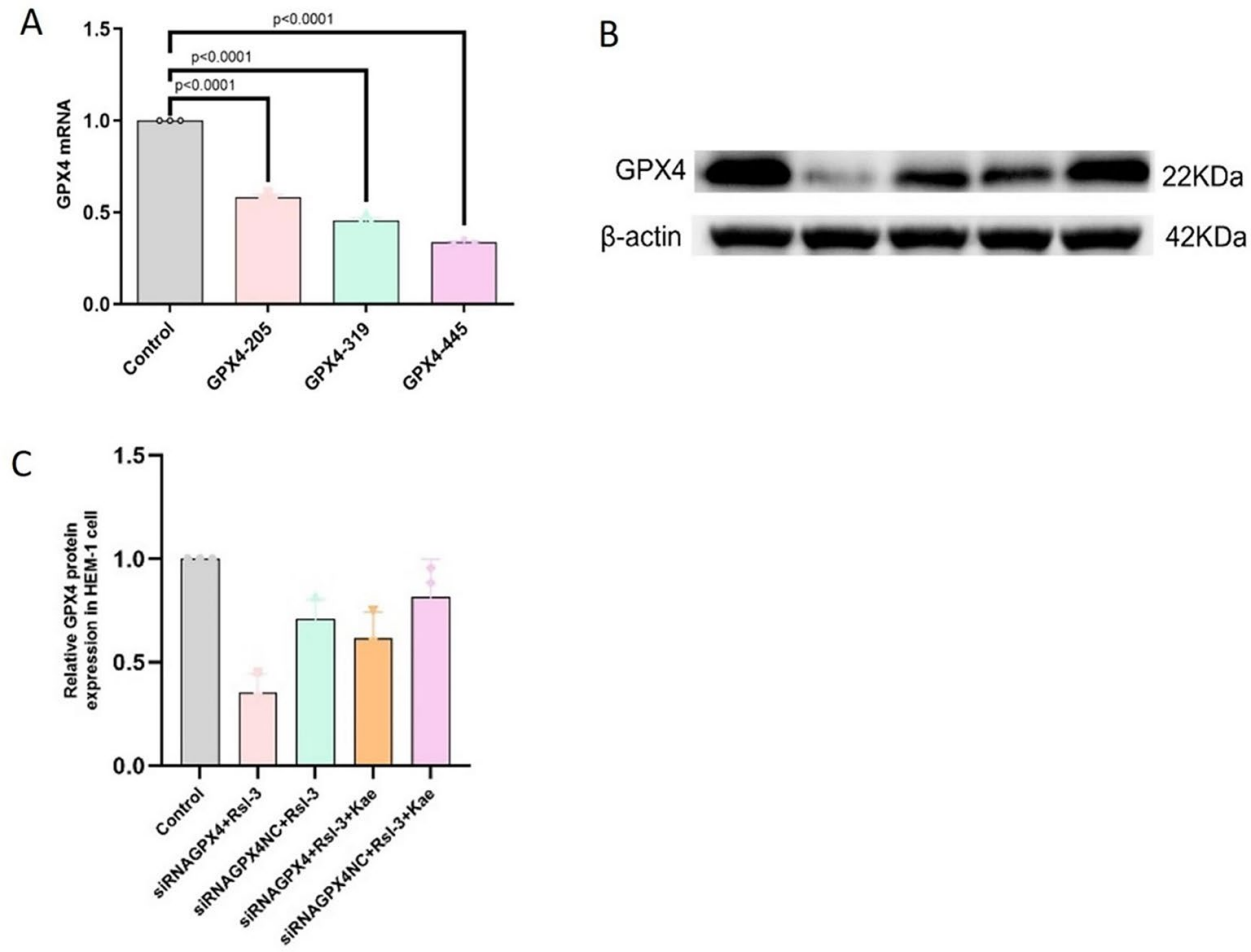
The expression of GPX4 was decreased after transfection. (**A**) The transfection effect was verified by the RT‒qPCR, and eventually, GPX4-445 was selected for subsequent experiments. (**B** and **C**) WB analysis of GPX4.

To clarify the effect of Kae on the ferroptosis of cells, the GPX4 gene was silenced and divided into five groups: the control group, the siGPX4 + RSL3 group, the siNC + RSL3 group, the siGPX4 + RSL3 + Kae group, and the siNC + RSL3 + Kae group. It was demonstrated that Kae had antagonistic effects on HEM-1 ferroptosis induced by RSL3 through the GPX4 ferroptosis signaling pathway. Firstly, the transfection effect was verified by the RT‒qPCR, and eventually, transfection plasmid siGPX4-445 was selected for gene silencing (Fig. 5A). Besides, the expression level of GPX4 was detected by WB to verify the transfection results. The WB results showed that the gray values of GPX4 in HEM-1 cells in the siGPX4 + RSL3 group were significantly lower than those in the siNC + RSL3 group (*P* < 0.0001). The gray-scale values of GPX4 in HEM-1 cells in the siGPX4 + RSL3 + Kae group were significantly lower than those in the siNC + RSL3 + Kae (*P* < 0.0001) (Fig. 5B-C).

**Fig. 6:**
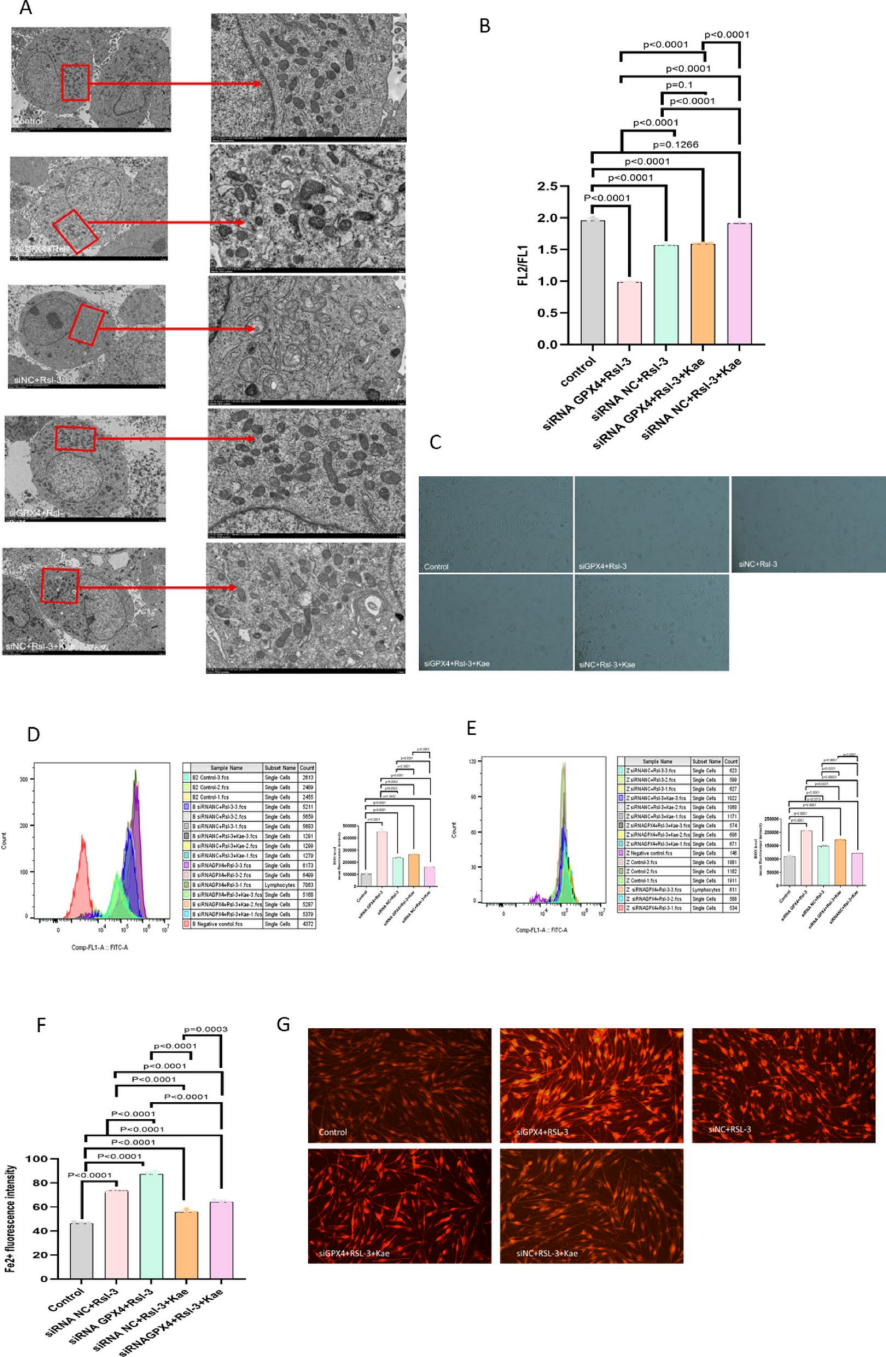
Suppression of GPX4 promoted melanocyte death. (**A**) After silencing the GPX4 gene, the mitochondrial morphological changes of HEM-1 were observed after the treatment with RSL3, Kae, or their combination. (**B**) The changes in the membrane potential were observed. (**C**) The number of HEM-1 in each group was observed under a microscope. (**D**) The production of intracellular ROS was detected by FC. (**E**) The production of lipid ROS was detected by FC. (**F**) After silencing the GPX4 gene, the changes in iron ions in HEM-1 after the treatment with RSL3 and Kae were observed. (**G**) The accumulation of iron ions in each group was detected under a fluorescence microscope.

Based on transmission electron microscopy, the mitochondrial morphological damage and mitochondrial membrane potential depolarization in the siNC + RSL3 group were significantly less than those in the siGPX4 + RSL3 group. Mitochondrial morphological damage and mitochondrial membrane potential depolarization in the siNC + RSL3 + Kae group were significantly reduced than those in the siGPX4 + RSL3 + Kae group (*P* < 0.0001) (Fig. 6A-B). Under an electron microscope, the amount of HEM-1 in the siGPX4 + RSL3 group was lower than that in the siNC + RSL3 group. The amount of HEM-1 in the siNC + RSL3 + Kae group was higher than that in the siGPX4 + RSL3 + Kae group (Fig. 6C). These results suggested that Kae can ameliorate mitochondrial damage and cell proliferation through the GPX4 ferroptosis signaling pathway.

### Kae reduced ROS and iron ion levels in RSL3-induced HEM-1 cells through the GPX4 ferroptosis signaling pathway

After the transfection with siGPX4, the ROS levels in five groups were measured. The experimental results showed that the levels of both intracellular and lipid ROS in the siGPX4 + RSL3 group were significantly higher than those in the siNC + RSL3 group (*P* < 0.0001), which further indicated that siGPX4 promoted RSL3-induced ferroptosis. Additionally, the levels of intracellular and lipid ROS in the siGPX4 + RSL3 + Kae group were significantly higher than those in the siGPX4 NC + RSL3 + Kae group, and siGPX4 reduced the protective effect of Kae on ferroptosis (*P* < 0.0001) (Fig. 6D-E). These results suggested that Kae exerted an antagonistic effect on RSL3-induced growth inhibition of HEM-1 cells through the GPX4 ferroptosis signaling pathway.

Meanwhile, the accumulation of iron ions in the cells was detected under a fluorescence microscope. The results showed that compared with the siRNA NC + RSL3 group, the siRNA GPX4 + RSL3 group exhibited brighter and more fluorescence, accompanied by the accumulation of a large amount of Fe^2+^. The siGPX4 + RSL3 + Kae group had more Fe^2+^ accumulation than the siRNA NC + RSL3 + Kae group (Fig. 6F-G). Therefore, it can be inferred that Kae may interact with iron ions via the GPX4 ferroptosis signaling pathway, thereby reducing intracellular and lipid ROS levels.

## Discussion

In this study, the network pharmacology and experimental verification results revealed that Kae can improve the mitochondrial morphology of HEM-1, reduce ROS production, and up-regulate the expression of the GPX4 ferroptosis pathway, thereby reducing the death of HEM-1. These results provided a laboratory basis for the treatment of vitiligo with Kae and a new potential drug for the treatment of this disease.

Network pharmacology studies have shown that a total of 490 potential targets and 13 key pathways, such as melanogenesis, ferroptosis, and tyrosine metabolism, were found in the treatment of vitiligo with Kae. Among them, the ferroptosis pathway was the sole pathway directly related to MC death. Previous research has also demonstrated that kae can inhibit ferroptosis in tubular epithelial cells, neuronal cells, hepatocytes, and other cell types, thereby ameliorating the associated diseases^2,19,18,20–24^ (Table 1). These results support the findings of network pharmacology studies.

**Table 1:** Researches of Kaempferol regulation of cell ferroptosis and treatment of vitiligo.

Article (references)	Target Cell	Disease	Signal Path
Chen J et al.^18^	Tubular epithelial cells ferroptosis	Renal inflammation and fibrosis	SLC7A11/GPX4
Yang et al.^5^	Neuronal ferroptosis	Neuronal injury	Nrf2/SLC7A11/GPX4
Li et al.^19^	Hepatocyte ferroptosis	Liver injury	Nrf2
Li et al.^20^	Liver cell ferroptosis	Liver damage	AMP-activated protein kinase
Kim et al.^21^	Human aortic smooth muscle cell ferroptosis	Vascular calcification	GPX4/ACSL4
Giri et al.^22^	H9C2 cardiomyocytes ferroptosis	Myocardial Ischemia Reperfusion Injury	Nrf2/GPX4
Xu et al.^23^	Ferroptosis of cardiomyocytes	Cardiovascular disease	Nrf2/GPX4/XCT
Xie et al.^24^	Melanocytes	Vitiligo	Inhibiting oxidative stress
Hu et al.^25^	HaCaT cell	Vitiligo	Inhibiting oxidative stress PI3K/AKT signaling
Zou et al.^26^	HaCaT cell	Vitiligo	Inhibiting oxidative stress
Lx et al.^27^	HaCaT cell	vascular calcification	Nrf2
Ran et al.^28^	Regulatory T-cells	Ischemia reperfusion injury	Nrf2/GPX4

Previous studies have reported some potential mechanisms of kae in the treatment of vitiligo and explored its potential in the treatment of vitiligo. Hu finded that kae increased HaCaT cell viability, reduced apoptosis, lowered ROS activity, effectively providing resistance against oxidative stress damage^25^. Zou verified that Kae can up-regulate the expression of Nrf2, thereby alleviating the oxidative stress damage of HaCaT cells induced by AAPH^26^. These two studies suggest that Kae may play a role in the treatment of vitiligo by regulating HaCaT cells. But, Xie found that kae can reduce oxidative stress damage in melanocytes and promote melanin synthesis^15^, this is similar to our findings. Our study also confirmed that kae reduces ROS production in melanocytes and we further investigated the mechanisms surrounding the ferroptosis pathway. These results provide a laboratory basis for the use of kae in the treatment of vitiligo.

However, there are some limitations in this study. Our team conducted exclusively in vitro experiments; however, the in vivo system is complex, necessitating further in vivo studies to elucidate the effects of Kae on vitiligo. Additionally, we identified several potential mechanisms by which Kae may treat vitiligo through network pharmacology and bioinformatics methods. However, we have only verified the mechanism related to “ferroptosis”; thus, further investigation into the other mechanisms is required. Besides, network-based predictions have certain limitations in accurately capturing complex cellular responses. Therefore, the effect and mechanism of kae in the treatment of vitiligo still need to be verified through more complementary experimental approaches. In the future, we aim to provide more substantial evidence for the efficacy of kae in treating vitiligo through in vivo studies, alternative models, or innovative methodologies.

## Conclusion

**Fig. 7:**
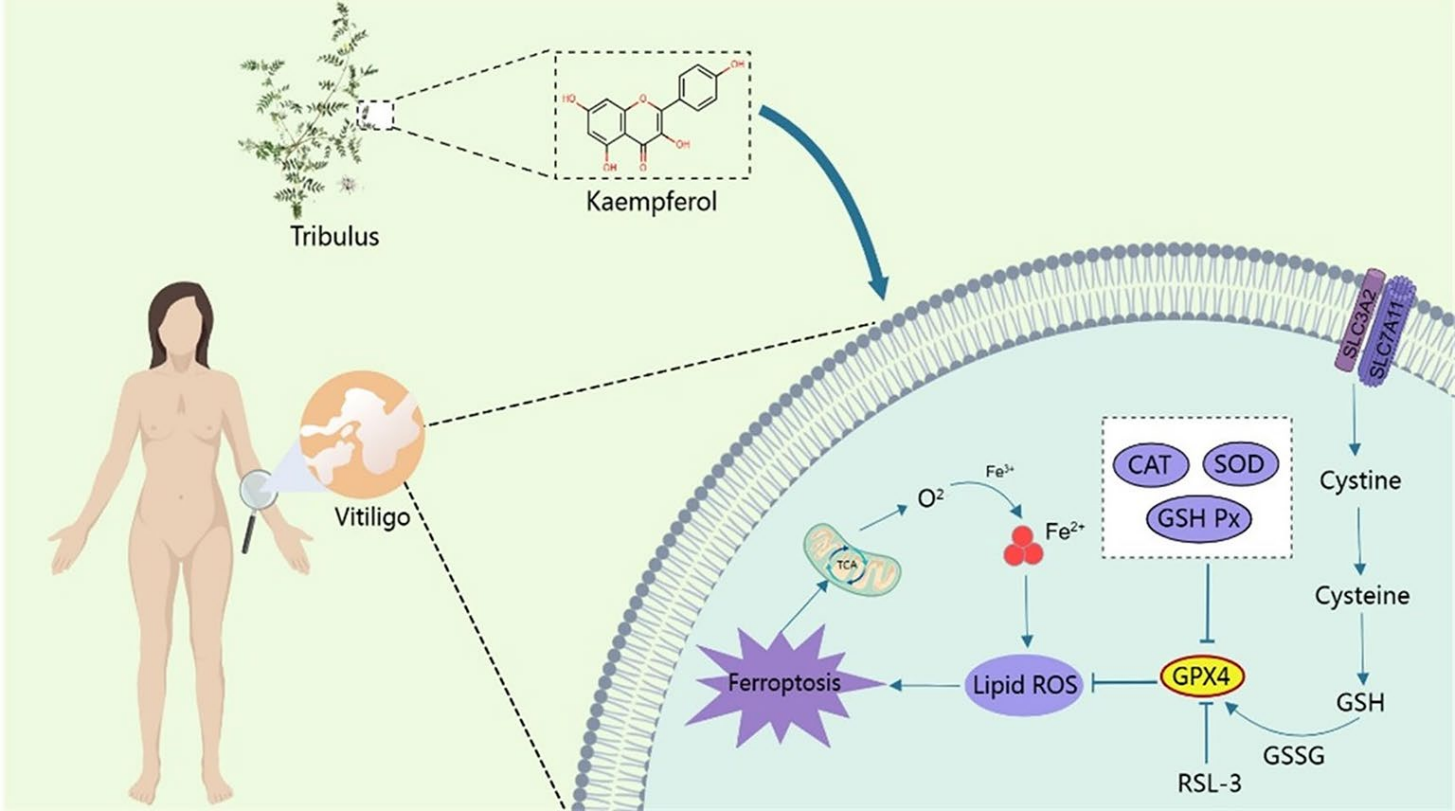
Kaempferol improves the mechanism of melanocyte death through the ferroptosis pathway.

Kae can upregulate the expression of the GPX4 protein within the ferroptosis pathway, thereby reducing MC death. It is a potential compound for the treatment of vitiligo (Fig. 7).

## Materials and methods

### Chemical structure and target information inquiry of Kae

Firstly, the SMILES ID and CAS number of Kae were obtained from PubChem (https://pubchem.ncbi.nlm.nih.gov/), and the potential targets of Kae were also collected. Then, the targets of Kae compounds were retrieved based on SwissTargetPrediction (http://www.swisstargetprediction.ch/), Comparative Toxicogenomics Database (CTD) (http://ctdbase.org/), BindingDB (http://bindingdb.org/bind/index.jsp), TargetNet(http://targetnet.scbdd.com/home/index/). Subsequently, the obtained targets were corrected and de-duplicated based on UniProt (https://www.uniprot.org/) and STRING (https://string-db.org/). Finally, the Wynn Diagram was plotted.

### Enrichment analysis of targets related to Kae effects

The enrichment analysis of the obtained drug targets was performed based on GO, DO, and KEGG with the aid of R. Based on that, the count value, the P-value, the adjusted P-value (the P-value corrected by multiple tests), and the enrichment plot were obtained^27–29^.

### Differential gene analysis of vitiligo

The search was performed based on the National Center for Biotechnology Information (NCBI) in GEO DataSets (https://www.ncbi.nlm.nih.gov). The search term was “vitiligo”, and the data with a sample size of more than 20 were downloaded to obtain genetic data on vitiligo and healthy subjects. Differential genes were analyzed to identify up-regulated and down-regulated genes in vitiligo patients and healthy subjects from the gene expression of the samples. Then, the differential genes were analyzed with the aid of R. The absolute logFC > 1 and the corrected P value < 0.05 were used to obtain differential genes. The top 20 differential genes with up-regulated and down-regulated expression, respectively, were selected to construct the volcano plot and heatmap.

### Gene set enrichment analysis of core genes related to vitiligo

Gene set enrichment analysis (GSEA) can be employed to elucidate the biological significance of characteristic genes using rapid GSEA R packages^30^. To achieve a standardized enrichment score for each analysis, 1,000 permutations of the gene set were performed. The “limma”, “org.Hs.eg.db”, “cluster Profiler” and “enrich Plot” packages were used to perform the GSEA enrichment analysis based on the GSE75819 dataset. The top 5 pathways with the most significant up-regulated and down-regulated expression in healthy subjects and vitiligo patients were screened according to FDR < 0.05.

### Weighted gene co-expression network analysis

Weighted gene co-expression network analysis (WGCNA) can be used to analyze gene expression patterns in multiple samples^31^. By clustering genes with similar expression patterns, the modules are divided by module eigenvalues, and the correlation between the modules and specific traits or phenotypes is analyzed. Therefore, the modules with a high correlation with traits are selected. In this study, abnormal samples were excluded by sample clustering. Then, a weighted co-expression network based on differential gene expression was constructed based on the “WGCNA” package in R. Subsequently, the power value of the weight parameter of the proximity matrix was determined, and then the TOM matrix gene cluster tree was constructed according to the power value. Next, a weighted co-expression network model was established to divide the integrated gene expression matrix into related modules. Finally, the module gene with the highest correlation with vitiligo was selected for further analysis.

### Drug-disease “MCODE” key cluster network and functional enrichment

The disease targets of vitiligo were used with the target genes of Kae, and the PPI of common targets between Kae and vitiligo was constructed based on STRING (https://string-db.org/cgi/input.pl) after duplicate genes were removed^32^. After the PPI network diagram was imported into Cytoscape, the centrality size of each gene in the protein-disease interaction network was calculated using the medium centrality of CytoNCA. Then, the size of the gene pattern was divided according to the centrality value, and the drug and disease genes were distinguished by different colors. Subsequently, the core subgroups in the DPPI network were screened using MCODE, and the genes with the highest score in the core subgroup were labeled as “keycluster” genes. Finally, the DO, GO, and KEGG enrichment analyses were performed on “keycluster” genes.

### Cell culture and treatment

HEM-1 (ATCC, PCS-200-012), after 2–5 passages, was cultured in the MC culture medium (Sciencell, 2201) containing the penicillin-streptomycin solution (Sciencell, 0503). HEM-1 was randomly assigned to 3 groups. Specifically, the first group was treated by the normal culture of HEM-1 (the control group); the second group was treated by HEM-1 that was cultured with RSL3 at 3µM for 24 h (the RSL3 group); the third group was treated by HEM-1 with the addition of RSL3 at 3µM for 24 h after 1µM Kae was added to HEM-1 for 2 h (the RSL3 + Kae group).

HEM-1 was transfected in accordance with the instructions of the PrimeScript RTreagent Kit (Vazyme, R323-01). Then, HEM-1 was randomly assigned to 5 groups: control group, siGPX4 + RSL3 group, siNC + RSL3 group, siGPX4 + RSL3 + Kae group, siNC + RSL3 + Kae group. Among them, the transfection time of siGPX4 or siNC by HEM-1 was 24 h. After transfection, the corresponding RSL3 or Kae was added as described above to interfere with HEM-1.

#### Cell counting kit-8 assay

The concentrations of RSL3 and Kae were detected by cell counting kit-8 (CCK-8) assay. The cells were seeded in 96-well plates at a density of 4 × 10^4^ cells per well, with 100 µl of culture medium added to each well. After a 24-hour incubation period, RSL-3 was introduced at an initial concentration of 10 µM, diluted by 1/2, gradient dilution of 10 Wells, and cultured for 24 h. At the end of the cell intervention, the CCK-8 reagent was added according to the manufacturer’s instructions and incubated at 37 °C for 2 h. Next, the optical density (OD) value was measured at 450 nm using an enzyme-labeler (Thermo Fisher Scientific, Waltham, MA, USA).

#### Mitochondrial morphology and membrane potential analysis

The effects of RSL3 and Kae on mitochondrial morphology of HEM-1 cells were observed in this experiment. Firstly, HEM-1 was incubated with 2.5% glutaraldehyde and then fixed with 1% osmic acid away from light. The mitochondrial ultrastructure was observed under a transmission electron microscope. The mitochondrial membrane potential was detected by the mitochondrial membrane potential assay kit (C2006, Beyotime, Shanghai, China). In brief, after the treatment for 24 h, the cells were re-suspended with the fresh medium, incubated with 0.5 mL of JC-1 solution at 37℃ for 20 min, centrifuged at 600 g and 4℃ for 3 min, and then washed twice with the JC-1 buffer. Finally, JC-1 monomers and JC-1 aggregates were detected by flow cytometry (FC) under excitation light at 490 nm and 525 nm, respectively.

#### Lipid and intracellular ROS detection

FC (Suzhou Saijing Biotechnology Co., LTD., A00-1-1102) was used to detect the level of ROS in HEM-1 cells and lipids after drug treatment. Intracellular ROS production was detected by CytoFLEX FC at an excitation wavelength of 502 nm (TexasRed labeling) and an emission wavelength of 530 nm (FITC labeling), respectively. After the cells were treated in the same manner, FC was used to detect the production of lipid ROS after the addition of the Image-iT™ Lipid Peroxidation Sensor with a concentration of 10µM and treated for 30 min. Fluorescence imaging was performed using conventional TexasRed^®^ (590 nm) and FITC (510 nm) emission filters.

### Detection of iron ions in HEM-1

According to the kit instructions, the 5µM FeRhoNoxTM-1 probe (GORYO CHEMICAL, GC901) was used to detect the Fe^2+^ content of cells in each group under a fluorescence microscope. After the cells were collected and digested, they were washed twice with Hanks’ balanced salt solution (HBSS), and 5µM FeRhoNoxTM-1 was added. Then, the cells were placed at 37℃ in a 5% CO_2_ incubator for 60 min and then washed three times with HBSS. Finally, the cells were observed and photographed under a fluorescence microscope.

### Western blot

After appropriate lysates and phenylmethylsulfonyl fluoride (PMSF) were added to the cells in each group for 30 min, the intermediate layer proteins were collected. Then, the protein concentration was detected by the bicinchoninic acid (BCA) protein concentration assay kit (Solarbio, Pc0020-500). Subsequently, the samples were collected and subjected to 10% sodium dodecyl sulfate–polyacrylamide gel electrophoresis (SDS-PAGE), and then they were transferred onto polyvinylidene fluoride (PVDF) membranes, followed by blocking with 5% fat-free milk in the tris-buffered saline with Tween (TBST). Next, the membranes were conjugated with primary antibodies targeting rabbit monoclonal anti-GPX4 (Abcam, ab125066), anti-TFR1 (Cell Signaling, 13113 S), and anti-β-actin (Proteintech, 66009-1) on a table concentrator at 4 °C overnight. After that, the membranes were rinsed by TBST three times and incubated with anti-mouse (Proteintech, SA00001-8) and anti-rabbit (Proteintech, SA00001-9) at room temperature for 1 h. After the film was placed in the image-shooting instrument, the enhanced chemiluminescence (ECL) luminescent solution was added. Finally, the image was taken, and the results were counted.

### Detection of SOD, CAT, and GSH-Px activities

The SOD test kit (Jian Chen Nan Jing A001-3), CAT kit (Addison, ADS-W-KY002), GSH-Px test kit (Nanjing) A005-1) were used to detect the activities of SOD, CAT, and GSH-Px of HEM-1 in each group according to relevant instructions.

### Real‑time quantitative polymerase chain reaction

Total RNA was extracted from HEM-1 cells by the TRIzol Reagent (Thermo, 15596-026). Approximately 1 µg of total RNA was reverse transcribed into cDNA by the PrimeScript RTreagent kit with gDNA Eraser (Vazyme, R323-01). The real‑time quantitative polymerase chain reaction (qPCR) was performed with the ChanQ Universal SYBR qPCR Master Mix (Vazyme, Q711-02) in the RT‒qPCR Detection System LC480 (Roche, Mannheim, Germany) for a total of 40 cycles (95^◦^C for 20 s, 60^◦^C for 30 s, and 72^◦^C for 20s). The expression of GPX4 relative to H-b-actin was analyzed based on the 2-△△Ct method. The primers are listed in Table 2.

**Table 2:** The primer sequence for RT-qPCR.

Primer	Forward sequence	backward sequence	bp	annealing temperature (℃)
H-b-actin	F:5’TGACGTGG ACATCCGCAAAG-3’	R:5’CTGGAAG GTGGACAGCGAGG-3’	204	56
GPX4	5’GTGTAACCAGTTCGGGAAGCAG 3’	5’GTTCCACTT GATGGCATTTCCC 3’	190	61

### Statistical analysis

The analysis method was selected based on whether the data met a normal distribution and the homogeneity of variance. When comparing data among multiple groups, one-way analysis of variance (ANOVA) was performed if the data were consistent with normal distribution (*P* > 0.05) and homogeneity of variance (*P* > 0.05); otherwise, Kruskal-Wallis test was used. When comparing between the two groups, if the data met the normal distribution (*P* > 0.05) and the variance was homogeneous (*P* > 0.05), the independent sample t test was performed; if the data met the normal distribution but the variance was not homogeneous, the t’ test was selected; if the data were not normal but the variance was homogeneous, the Mann-WhitneyU test was selected. IBM SPSS 20.0 was used for statistical analysis, and *P* < 0.05 was considered statistically significant (bilateral test). Graphs were processed using GraphPad Prism 9.5.

## Electronic supplementary material

Below is the link to the electronic supplementary material.

Supplementary Material 1

## Data Availability

Correspondence and requests for materials should be addressed to J.M.L.
