# Author Correction: An investigation into the mechanism for Kaempferol improving melanocyte death based on network Pharmacology and experimental verification

**DOI:** 10.1038/s41598-025-19323-w

**Published:** 2025-09-30

**Authors:** Jinming Li, Yeqiang Song, Meng Yang

**Affiliations:** 1 Department of Dermatology, Shandong Provincial Third Hospital, Jinan, 250000 China; 2 Cosmetic dermatology, Affiliated Hospital of Shandong University of Traditional Chinese Medicine, Jinan, 250000 China

Correction to: *Scientific Reports* 10.1038/s41598-025-91905-0, published online 12 March 2025

The original version of this Article contained an error in Figure 6, where in panel G, the siNC + RSL3 group and the siNC + RSL3 + Kae group were duplicated due to an error in combining the images.

**Fig. 6:**
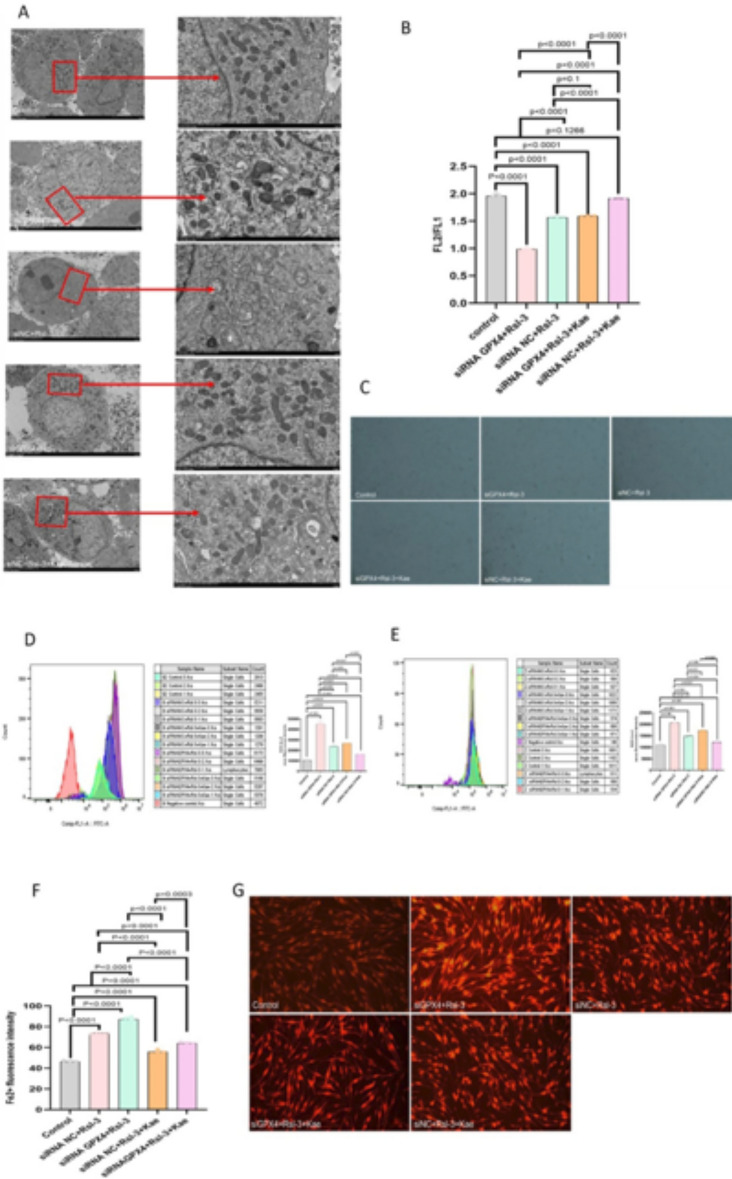
Suppression of GPX4 promoted melanocyte death. (**A**) After silencing the GPX4 gene, the mitochondrial morphological changes of HEM-1 were observed after the treatment with RSL3, Kae, or their combination. (**B**) The changes in the membrane potential were observed. (**C**) The number of HEM-1 in each group was observed under a microscope. (**D**) The production of intracellular ROS was detected by FC. (**E**) The production of lipid ROS was detected by FC. (**F**) After silencing the GPX4 gene, the changes in iron ions in HEM-1 after the treatment with RSL3 and Kae were observed. (**G**) The accumulation of iron ions in each group was detected under a fluorescence microscope.

The original Article has been corrected.

