# Supplementary material for: An investigation into the mechanism for Kaempferol improving melanocyte death based on network Pharmacology and experimental verification
Source: Sci Rep. 2025 Mar 12;15:8616. doi: 10.1038/s41598-025-91905-0 (PMC11904206; doi:10.1038/s41598-025-91905-0)

First time actin-22kDa

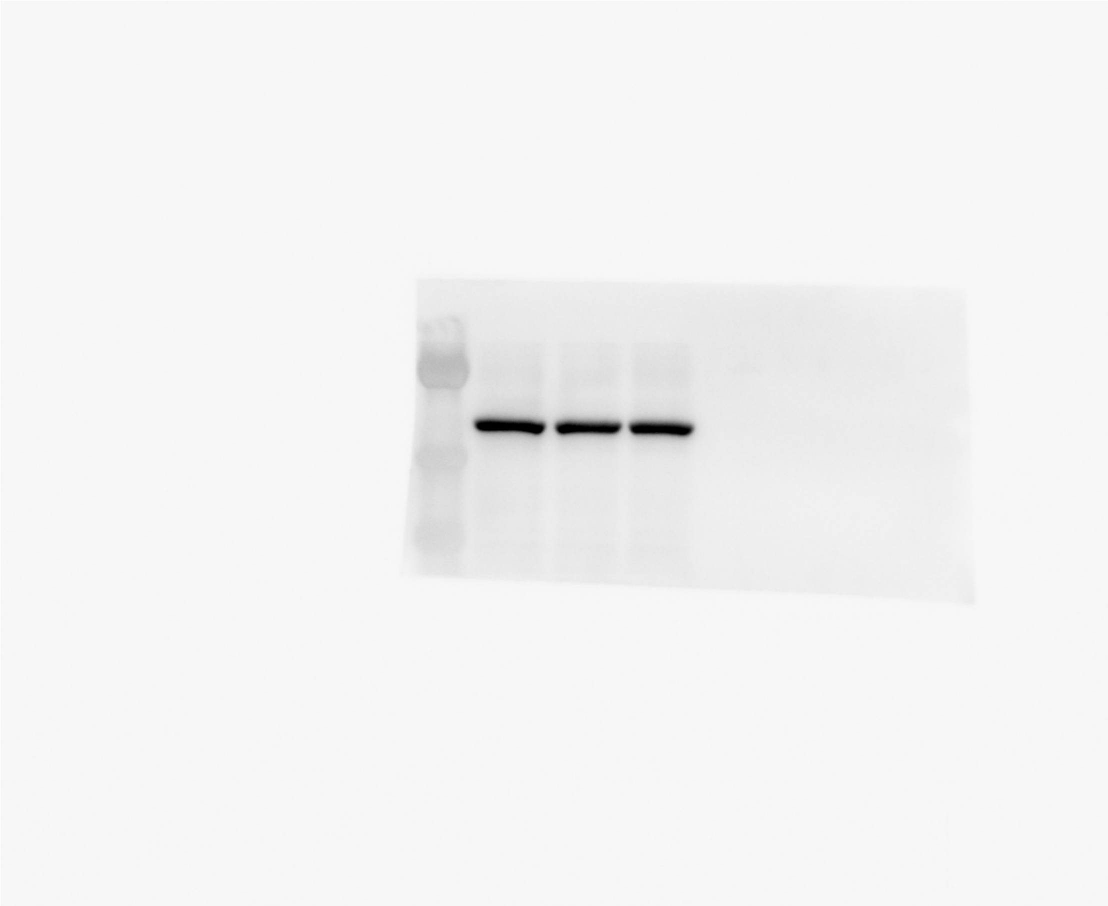

Second time actin-22kDa

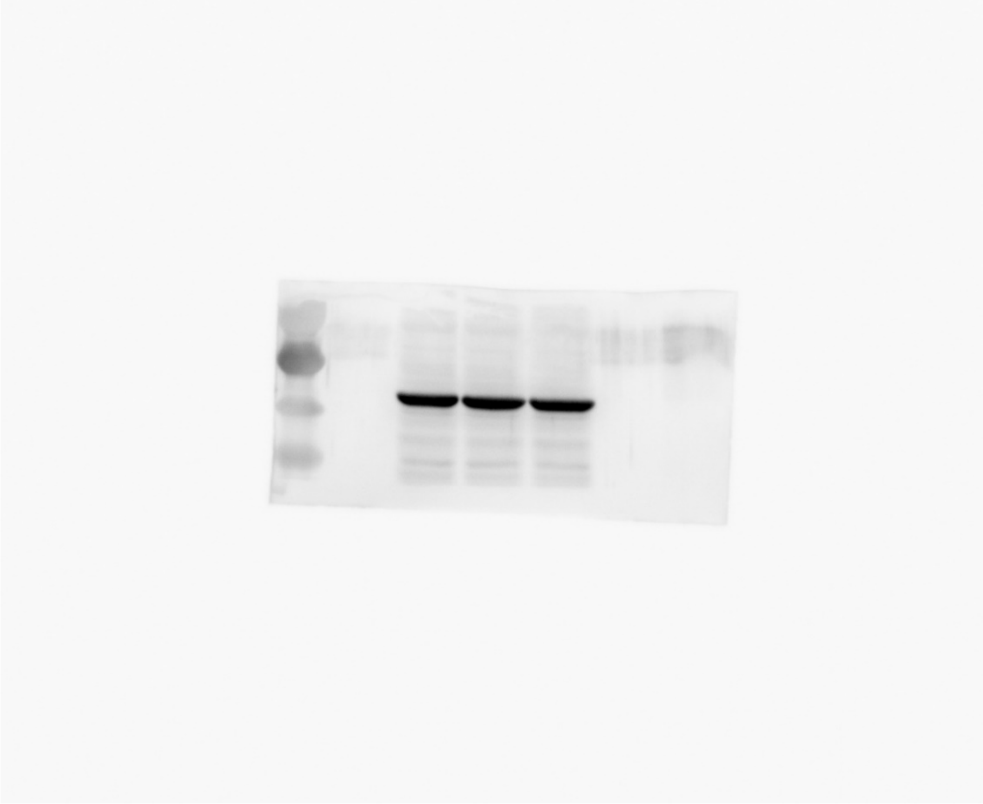

Third time actin-22kDa

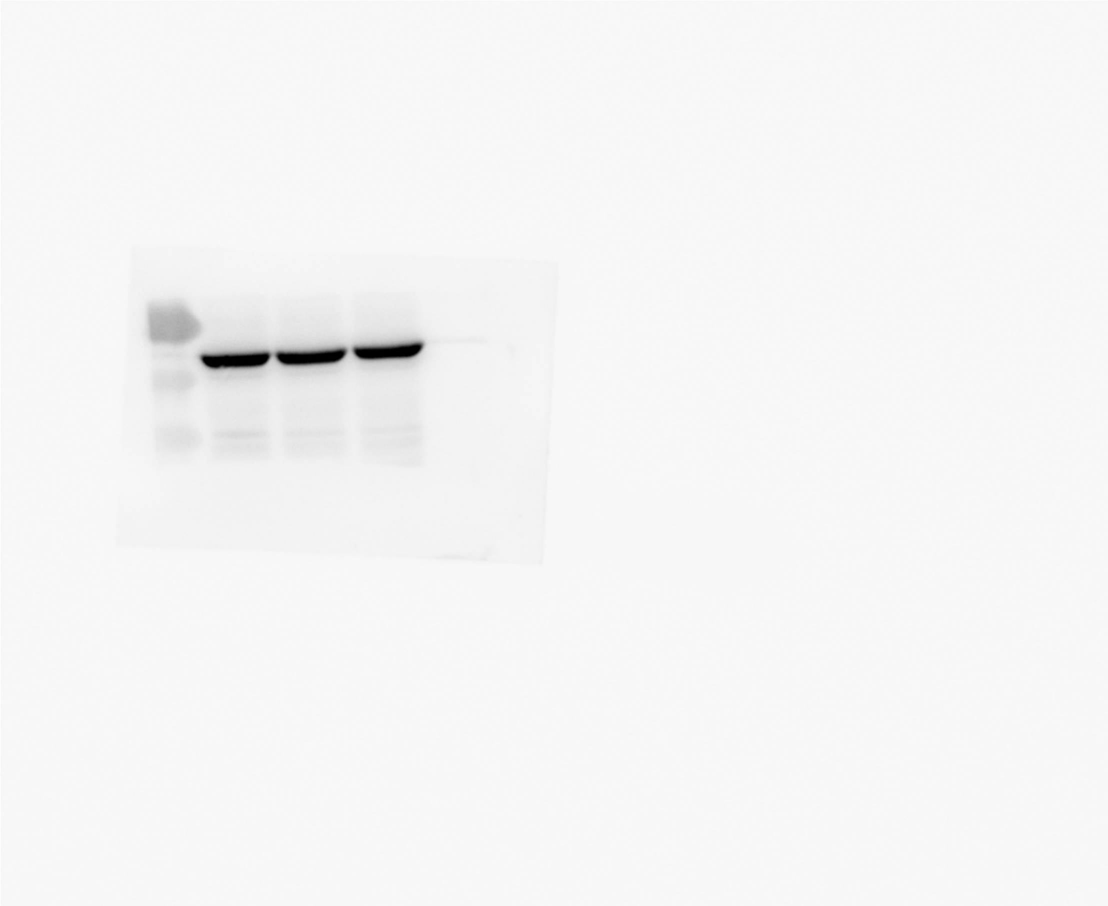

First time GPX4-42kDa

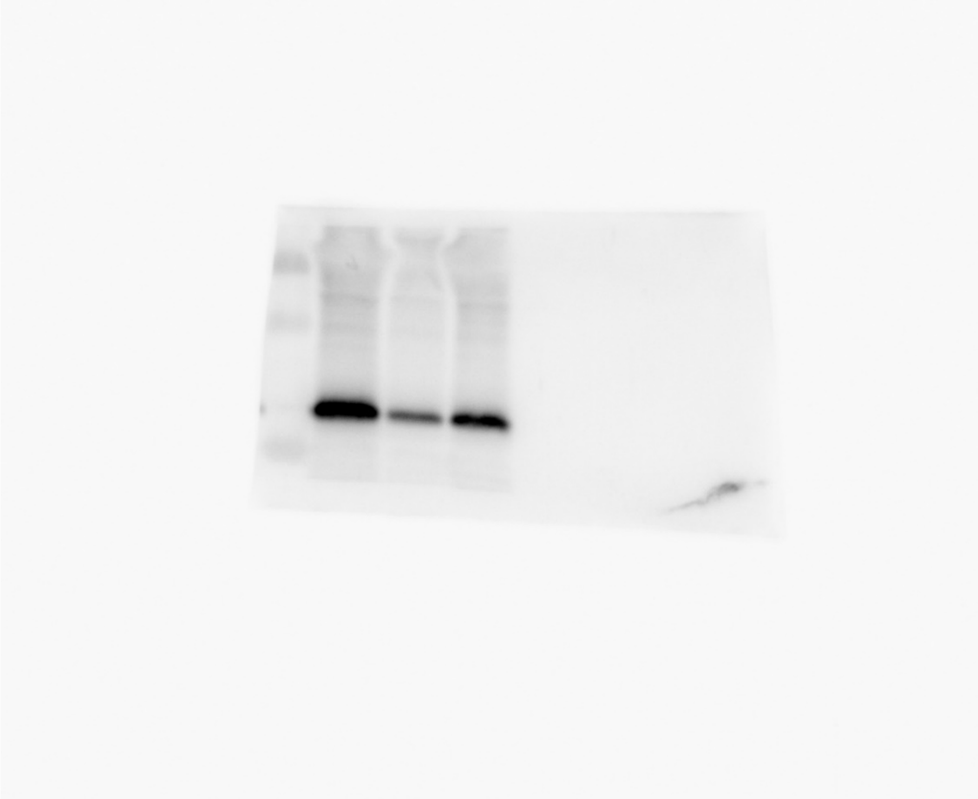

Second time GPX4-42kDa

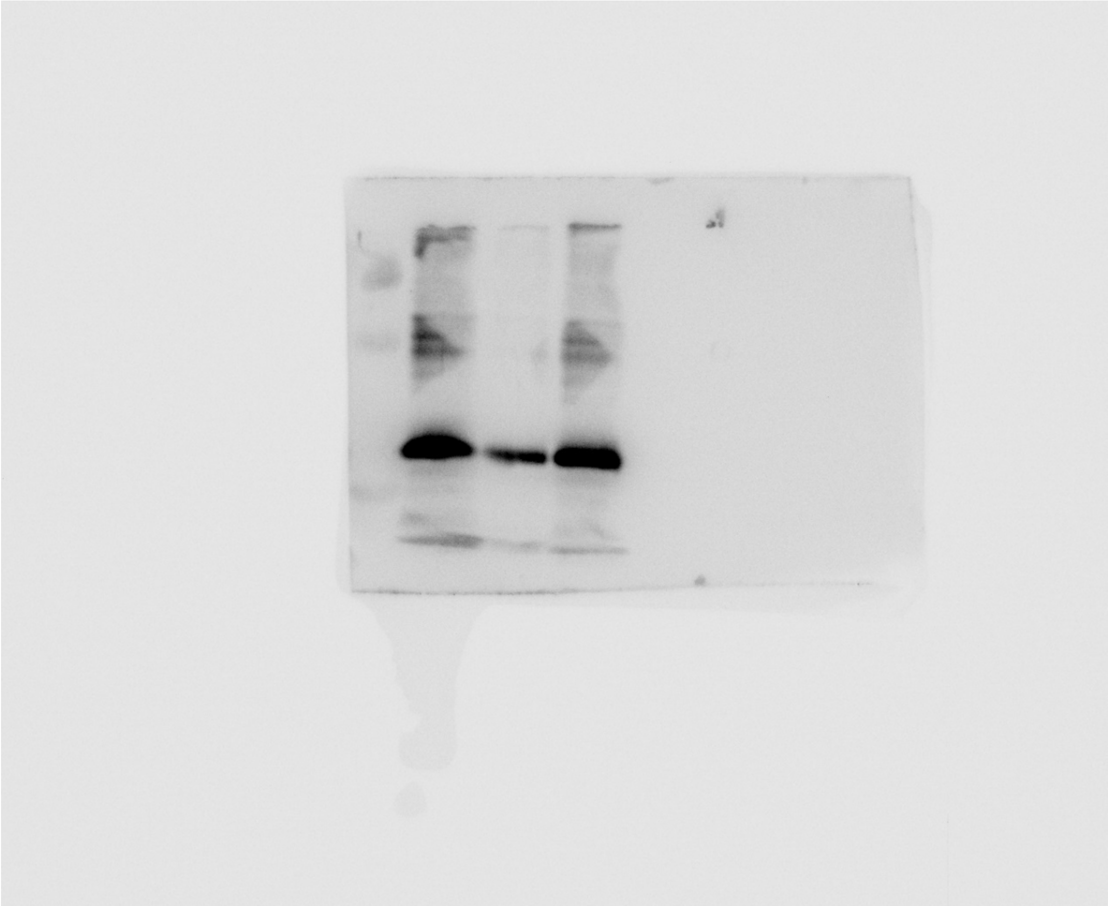

Third time GPX4-42kDa

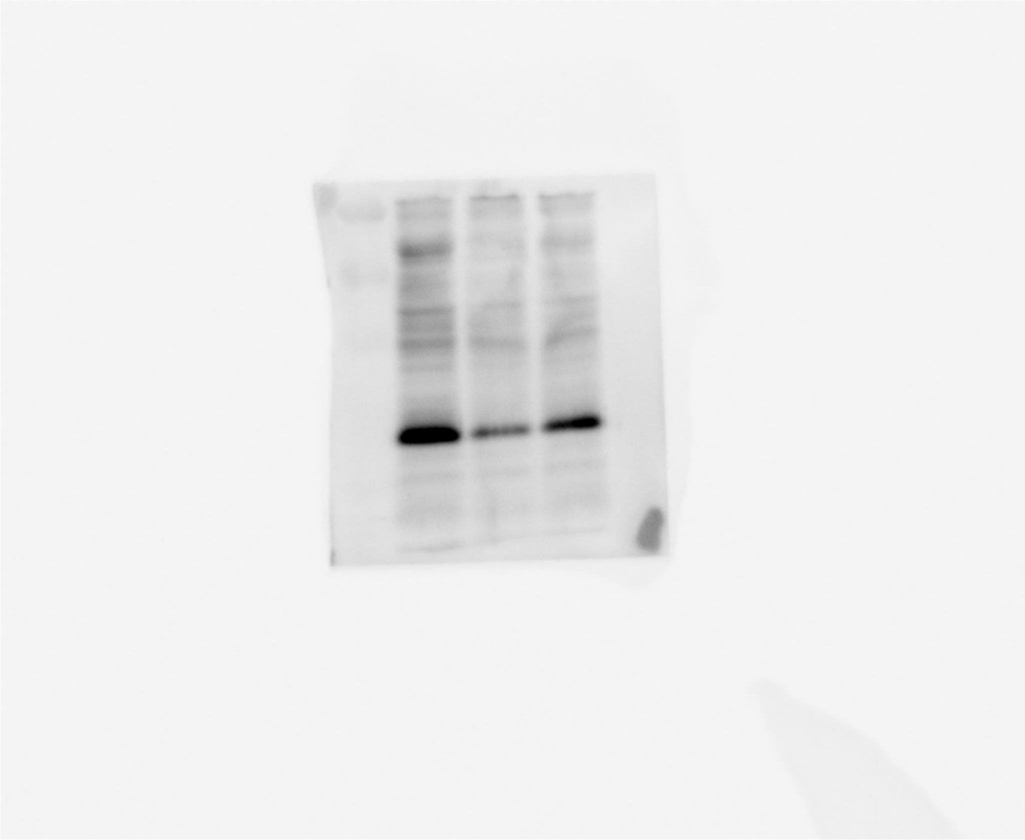

First time actin-22kDa

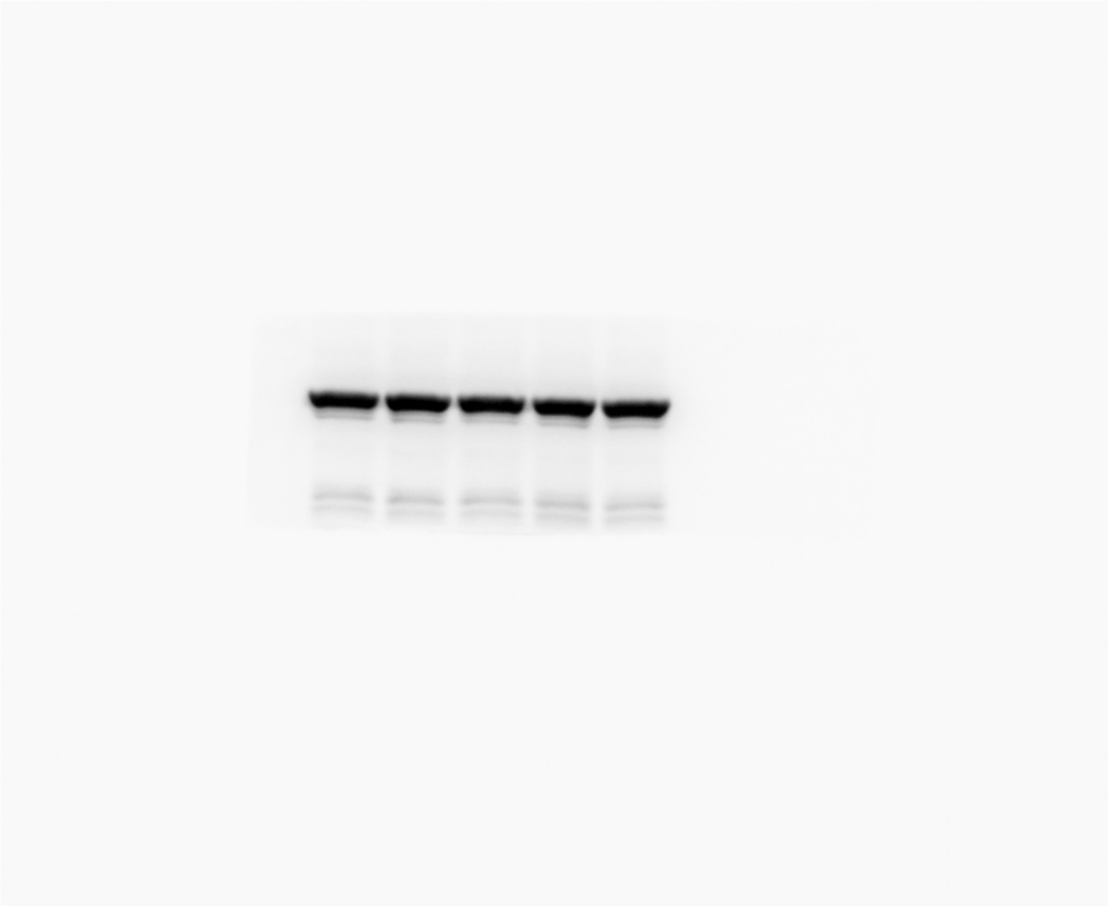

Second time actin-22kDa

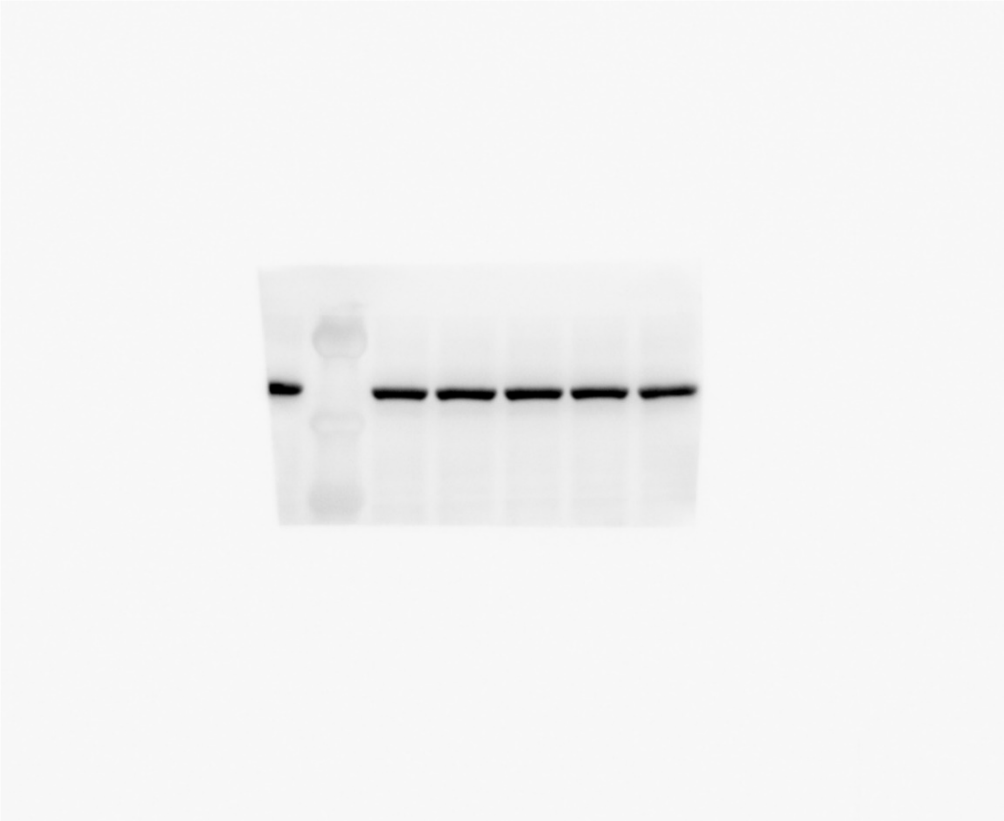

Third time actin-22kDa

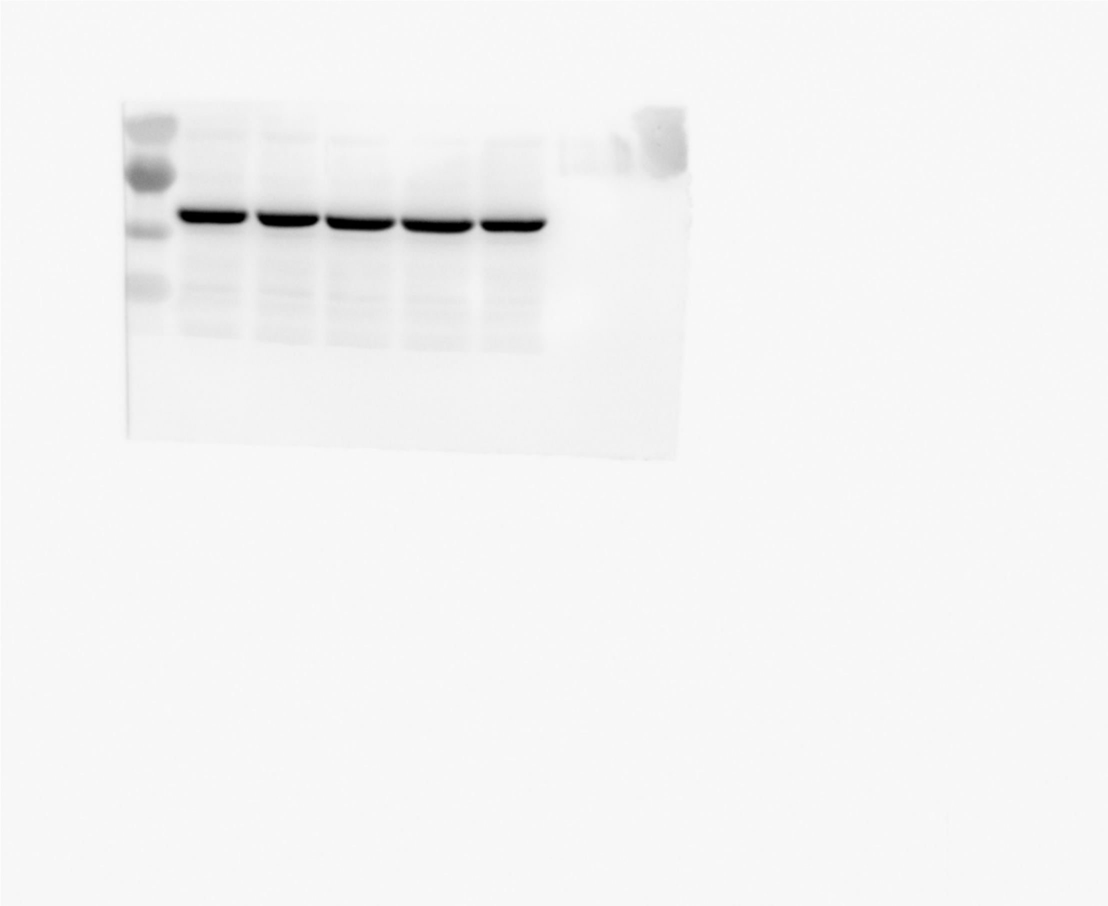

First time GPX4-42kDa

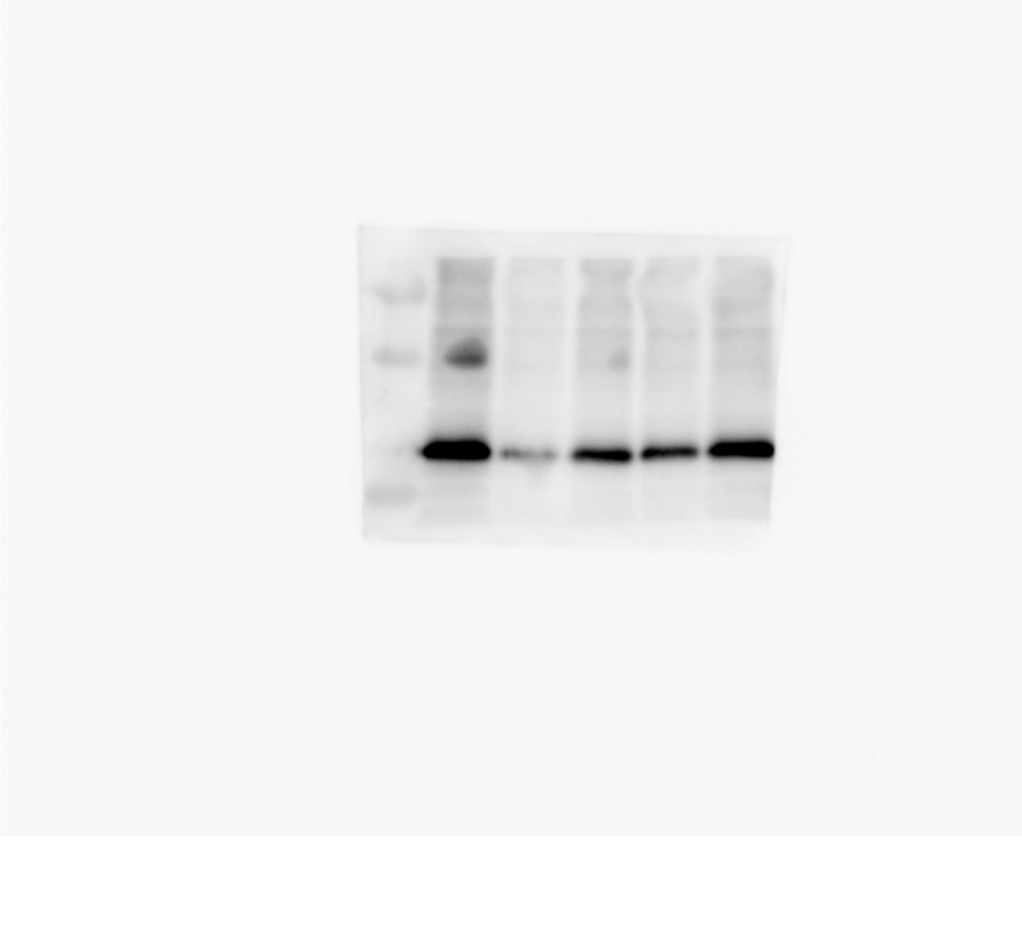

Second time GPX4-42kDa

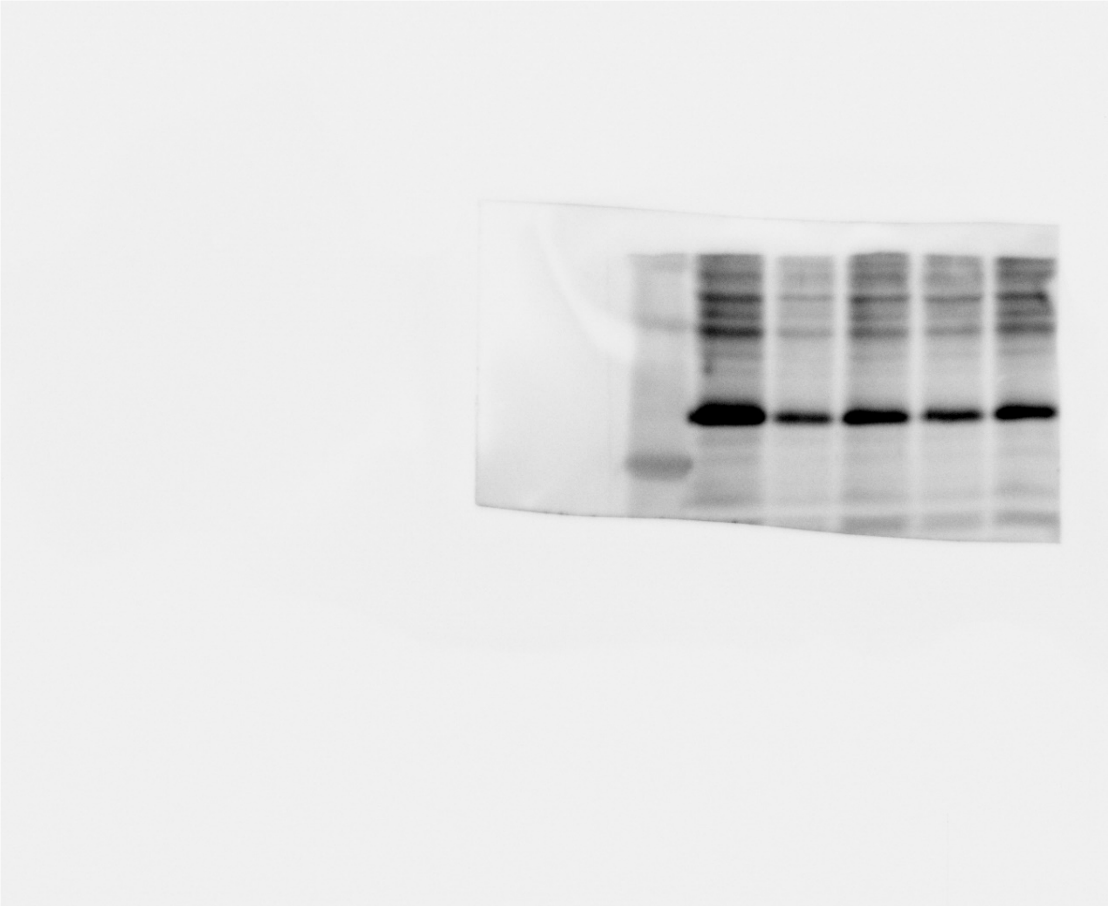

Third time GPX4-42kDa

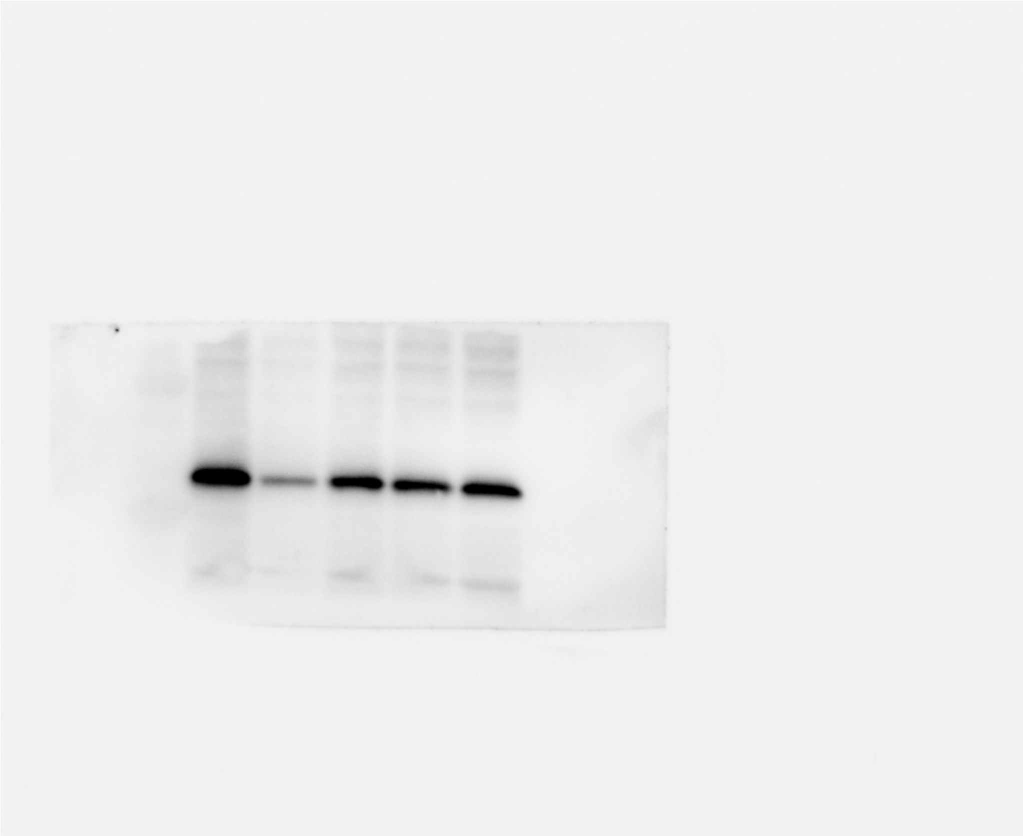

Supplement: Supplementary file 1 — Supplementary Material 1 [file 41598_2025_91905_MOESM1_ESM.pdf]
